# Autophagy and Noroviruses

**DOI:** 10.3390/v11030244

**Published:** 2019-03-12

**Authors:** Kevin Furlong, Seungmin Hwang

**Affiliations:** 1 Committee on Microbiology, The University of Chicago, Chicago, IL 60637, USA; furlongk@uchicago.edu; 2 Committee on Immunology, The University of Chicago, Chicago, IL 60637, USA; 3 Committee on Cancer Biology, The University of Chicago, Chicago, IL 60637, USA; 4 Department of Pathology, The University of Chicago, Chicago, IL 60637, USA

**Keywords:** norovirus, positive-sense RNA virus, autophagy, LC3 conjugation, replication compartment, interferon-inducible GTPases

## Abstract

Autophagy is an essential cellular process by which a cell degrades materials within its cytoplasm. Intracellular pathogens like viruses must deal with autophagy, either positively or negatively, for their own survival and replication. For some viruses, autophagy can even play proviral roles, helping their replication or dissemination. For other viruses, including noroviruses, the exact role of autophagy is more complex. This short review seeks to summarize the known interactions between autophagy, autophagy proteins and norovirus, and to address remaining questions relevant to these interactions.

Successful viral replication requires a complex series of interactions within a permissive host cell; a virus needs to co-opt many host proteins for its own use, while also evading immune sensors and effectors that would otherwise lead to its eradication [1]. Thus, understanding the role of host factors in the life cycle of a virus can be used to develop new strategies to impede viral replication [2]. Recent studies have shown that proteins initially characterized for their role in autophagy also play important roles in the life cycle of several viruses [3]. The interaction between autophagy and murine norovirus (MNV) is of particular interest, as it reveals a previously unexplored function of autophagy proteins in the control of intracellular pathogens in general, as well as functions that are not related to their capacity to facilitate degradative autophagy [4].

Autophagy is essentially a homeostasis process by which cellular materials are delivered to, degraded in, and recycled back from lysosomes via the cooperation of several autophagy-related (ATG) proteins [5,6]. Originally described as “bulk segregation and digestion of portions of the cytoplasm” [7], autophagy has since been implicated in several different types of intracellular homeostatic maintenance, ranging from the recycling of dysfunctional organelles to the control of intracellular pathogens [8,9]. On a larger scale, autophagy is also known to play critical roles in the pathogenesis of a wide-variety of human impairment including ageing, inflammatory bowel disease, and several cancers [8]. The term “autophagy” itself contains several related yet distinct processes; microautophagy, chaperone-mediated autophagy (CMA), and macroautophagy. As neither microautophagy, which involves direct uptake of bulk cargo by lysosomes [10], nor CMA, which degrades a small subset of proteins via recognition of a specific protein tag [11], have been implicated in host-pathogen defense against noroviruses, macroautophagy will primarily be discussed here.

Macroautophagy (hereafter, “autophagy”) is a process by which material to be degraded is enclosed within an extension of the membrane, thought to be derived from host endoplasmic reticulum (ER) [12,13]. This structure, known as an autophagosome, is formed through conjugation of microtubule-associated protein 1 light chain 3 (LC3) or its homologs to membranes [14]. The autophagic process itself (diagrammed in Reference 8) can be induced in response to several different stimuli, including nutrient deprivation, ER stress, various immune signals, and activation of certain cell surface receptors [15,16]. Following induction, the first of three major complexes known as the initiation complex (ULK1 (unc-51 like kinase 1)-ATG13-FIP200 (focal adhesion kinase family interacting protein of 200 kD)-ATG101 complex) translocates to the ER and triggers the activation of the second complex. This complex, the autophagy-specific class III phosphatidylinositol-3-OH kinase complex (nucleation complex) is activated in part via interaction with the ER-localized protein vacuole membrane protein 1 (VMP1) [16,17,18,19]. Consequent formation of phosphatidylinositol-3-phosphate (PtdIns(3)P) recruits ER localized, yet dispersed double FYVE containing protein 1 (DFCP1) and WD-repeat protein interacting with phosphoinositides (WIPIs), which are partially responsible for localized reshaping of the ER membrane [17]. Dynamic interactions among these molecules begin the nucleation of the early autophagosome [17,18,19]. The final step of the autophagosome formation involves increasing membrane curvature of the nascent autophagosome via a third protein complex known as the ATG12–ATG5-ATG16L1 complex, also called the elongation complex [20]. This E3-like ligation complex requires its associated E1-like activator and E2-like conjugator to function. The covalent ATG12–ATG5 conjugate must first be formed by ubiquitin-like conjugation of ATG12 to ATG5 through the E1-like ATG7 and the E2-like ATG10 [21]. The resulting ATG12–ATG5 conjugate complexes with the as of yet independent ATG16L1 to form the ATG12–ATG5-ATG16L1 complex. This complex conjugates another ubiquitin-like LC3 and its homologs to phosphatidylethanolamine (PE) in the membrane bilayer of the growing autophagosome, following the function of ATG7 and ATG3 as E1-like and E2-like enzymes, respectively [21,22]. The mammalian homologs of LC3, namely gamma-aminobutyric acid receptor associated protein (GABARAP), GABARAP-like 1 (GABARAPL1), GABARAPL2 (also known as Golgi-associated ATPase enhancer of 16 kDa or GATE16), are a series of proteins sharing high sequence similarity that function in autophagy through recognition of specific cargoes [22,23]. The growing autophagosome eventually encloses the material to be degraded and fuses with the lysosome. Proteomic analysis of individual members of the initiation, nucleation, and elongation complexes as well as LC3 homologs have shown significant interaction among members of each, suggesting a much more complex and dynamic formation pattern that is not currently well understood [24]. 

Characterization of interactions between RNA viruses and these proteins have demonstrated both proviral and antiviral roles for autophagy [9]. Poliovirus infection of human cells induces formation of double-membraned vesicles (DMVs), upon which viral replication takes place and which resemble autophagosomes. [25]. Treatment of the infected cells with known chemical inducers of autophagy increases production of extracellular virions. Conversely, treatment with an inhibitor of autophagy decreases such production, suggesting a role for autophagy in release of the virus [25,26]. Studies of the interaction between autophagy and H5N1, a highly pathogenic strain of influenza A virus (IAV), show an accumulation of autophagosomes in individual mice and humans who succumb to the virus. Prophylactic treatment to prevent viral manipulation of autophagy signaling increases survival of infected mice, while decreasing the associated lung damage [27]. Separate study of autophagy and influenza shows a role for a viral protein, Matrix 2 (M2), in relocating LC3 from the cytoplasm to the plasma membrane, thereby subverting autophagy-machinery for virion stability [28]. Chemical and genetic inhibition of autophagy is also known to suppress the replication of several other RNA viruses including hepatitis C virus (HCV), coxsackievirus B3 (CVB3), and Japanese encephalitis virus (JEV) [26,29,30,31]. On the contrary, autophagy can mediate the suppression of HCV replication via direct autophagosomal degradation of the viral nonstructural 5A (NS5A) protein [32]. In general, the formation of autophagosomes is proviral for a wide range of distinct RNA viruses, either directly harboring replicating viruses, or indirectly via mechanisms that are not fully understood. In contrast, studies with vesicular stomatitis virus (VSV) have shown autophagy-mediated sensing of viral infection via delivery of viral nucleic acids to Toll-like receptor (TLR)-containing endosomes, suggesting a broad antiviral role for autophagy [33,34]. Collectively, autophagy plays significant and specific roles in the life cycle of many viruses.

Recent studies have also shown roles for several autophagy proteins in MNV replication specifically. Such an interaction was revealed upon the study of MNV replication in *Atg5*-deficient cells, lacking the elongation complex and thus the entire autophagy pathway. The replication of MNV is comparable in bone marrow-derived macrophages (BMDMs) from *Atg5*^flox/flox^ and *Atg5*^flox/flox^ + *LysMCre* mice. In contrast, activation of the macrophages with interferon-gamma (IFNG) inhibits MNV replication in BMDMs from *Atg5*^flox/flox^ mice, but not in the ones from *Atg5*^flox/flox^ + *LysMCre* mice [35]. *Atg5*^flox/flox^ + *LysMCre* mice are also significantly more susceptible to MNV infection than *Atg5*^flox/flox^ mice, in the absence of type I IFN receptor (*Ifnar*^−/−^) signaling [35], suggesting an essential role of *Atg5* in the IFNG-mediated control of MNV infection in vivo as well as in vitro. Similarly, the E1-like activity of ATG7 and the E2-like activity of ATG3 in the LC3 conjugation system are required for this IFNG-mediated control of MNV infection, as expression of an enzymatically inactive form of either of these in macrophages lacking the WT form are no longer able to control the infection. In contrast, cells from mice lacking *Ulk1*, *Ulk2*, or *Atg14*, i.e. without the initiation complex or the nucleation complex, can control MNV infection similarly to WT macrophages upon activation by IFNG [4]. Intriguingly, IFNG inhibits the replication of MNV in an Atg5-dependent manner at the formation step of the MNV replication compartment (RC), which are structures formed during infection with multiple positive-sense RNA viruses, and where viral genome replication takes place. Moreover, the ATG12–ATG5-ATG16L1 complex itself can localize at the MNV RC. Taken together, it is clear that the ATG12–ATG5-ATG16L1 complex is required for the IFNG-mediated control of MNV outside the context of canonical autophagy.

The requirement of the LC3 conjugation machinery implicates LC3 homolog conjugation as a major determinant of MNV control. This same machinery has been linked to the IFNG-mediated control of *Toxoplasma gondii*, a protozoan intracellular parasite. *T. gondii* infection involves active invasion of a host cell and self-compartmentalization in a nonfusogenic membranous structure derived from the host plasma membrane, known as parasitophorous vacuole (PV) [36]. To combat *T. gondii*, the LC3 conjugation system of autophagy functions in a noncanonical role [37,38,39]. Specifically, the conjugation system is required to recruit two families of IFN-inducible dynamin-like GTPases: immunity related GTPases (IRGs) and guanylate-binding proteins (GBPs) to the PV, vesiculating and disrupting the PV and eventually killing the pathogen. Importantly, neither chemical nor genetic inhibition of the initiation complex or nucleation complex, essential components of canonical autophagy, have any effect on control of *T. gondii* in this manner [37,40,41]. This targeting of anti-microbial effectors to pathogen-containing vacuoles is now known as “Targeting by AutophaGy proteins” (TAG) [40,41].

Similar to *T. gondii*, there is evidence of IFNG-mediated IRG and GBP recruitment to the MNV RC. Knockout of almost all known LC3 homologs (both alleles of *Lc3a*, *Lc3b*, *Gabarapl1*, and *Gabarapl2*, and a single allele of *Gabarap*) in mouse embryonic fibroblast (MEF) cells reveals the necessity of those LC3 homologs for the control MNV by IFNG. Furthermore, localization of both LC3 and the IFN-inducible GTPases to the MNV RC has been shown via colocalization of immunity-related GTPase a6 (IRGA6), a representative member of the IRG family, and several GBP family members with endogenous LC3 at the MNV RC. Such localization of LC3 and the GTPases to the MNV RC requires *Atg5* but not *Atg14*, consistent with the TAG mechanism. Indeed, MNV replication is not efficiently controlled by IFNG in BMDMs from mice lacking functional IRG or GBP systems (*Irgm1*^−/−^*Irgm3*^−/−^ and *Gbp*^chr3−/−^, respectively). Consistently, MNV replication is significantly higher in these mice compared to WT mice [4]. While the in vivo evidence for the requirement of all LC3 homologs is lacking, the unique necessity of *Gabarapl2* was shown for the proper recruitment of the GTPases to the *T. gondii* PV in a knockout mouse model [42]. The exact roles of each of the LC3 homologs and IFN-inducible GTPases is not yet known, but it is clear that only the LC3 conjugation system of autophagy and the IFN-inducible GTPases are required for IFNG-mediated control of MNV [4].

A major missing link in the TAG system relating autophagy proteins to MNV resistance is how the ATG12–ATG5-ATG16L1 complex is involved in sensing the MNV RC and recognizing it as a target for destruction. In this regard, it is noteworthy that ATG16L1 localizes at the MNV RC, even in the absence of either ATG5 or ATG7, suggesting a key role of ATG16L1 in such sensing of the MNV RC [4]. Another crucial question is the exact manner by which the IFN-inducible GTPases would break down the MNV RC. The *T. gondii* PV is large enough to be vesiculated by the GTPases into small vesicles out of the PV membranes [43,44,45]. However, the MNV RC itself is close to the size of the individual vesicles removed from the PV to break it down [37,46]. Assuming that the same pattern of vesiculation cannot take place with such a comparatively smaller target, further investigation is needed to determine exactly how the IFN-inducible GTPases interact with the MNV RC such that it inhibits viral replication (detailed discussion in [39]).

Other recent work into the intersection between MNV and autophagy has shown that, regardless of any potential antiviral role, MNV infection may result in an increase in autophagy [47]. Both a shift toward punctate LC3 as well as an increase in autophagosome-like DMVs are visible at 12 hours post infection in the RAW264.7 macrophage cell line by MNV–common signs of autophagy and MNV replication. Conversely, colocalization of LC3 and lysosomal-associated membrane protein 1 (LAMP1), indicative of successful fusion of autophagosomes with lysosomes, is significantly lower in MNV infected cells compared to those experiencing chemically-induced autophagy. This, along with the accumulation of autophagy receptor/cargo p62 (SQSTM1), indicates that while autophagy may be activated in response to infection, degradation of autophagosomal cargo via fusion with lysosomes is inhibited [47]. It remains unclear whether this increase in autophagy results from an attempt by the virus to co-opt the autophagy system, or from the host attempting to combat the virus.

In addition to these intracellular interactions, organism-wide interactions between autophagy proteins and norovirus is also an area of interest. Both the morphology and transcriptional profile of Paneth cells in mice hypomorphic for ATG16L1 expression (ATG16L1HM) change dramatically, showing an increase in pro-inflammatory cytokine transcription and lipid metabolism. A genome wide association study (GWAS) identified a common mutation in human ATG16L1 that is associated with an increased risk for Crohn’s disease in humans [48,49,50]. Crohn’s disease patients homozygous for the mutant allele display a variety of gastrointestinal abnormalities, including differences in Paneth cells similar to those seen in ATG16L1HM mice [51]. Strikingly, attempts to rederive ATG16L1HM mice with such an altered Paneth cell phenotype in a different environment initially failed, but infection of these mice with a chronic strain of MNV, MNV.CR6 (CR6) restored the altered transcriptome, unique Paneth cell morphology, and abnormal granule packaging [52]. Importantly, this interaction extended beyond Paneth cells, as detection of viral titers in distinct cell types showed that the Paneth cells themselves are not infected. Additionally, ATG16L1HM mice produce virus at the same rate as littermate control mice, suggesting that simple change in viral titer is not responsible for this stark phenotypic difference.

Both the ATG16L1HM genotype and MNV infection are required to achieve the Crohn’s disease-like phenotype. This required interaction between a genetic and infectious element has been called the “virus-plus-susceptibility gene” interaction. RNA-seq analysis of infected ATG16L1HM mice also showed that groups of genes were altered not only to different magnitudes, but also in different directions. That is, transcriptional shifts of genes were not simply exacerbated in the virus-plus-susceptibility model in response to the same stimulus; they were completely different, indicating a total shift in the cellular response to infection depending on the presence of the ATG16L1HM genotype. Beyond transcriptional differences, ATG16L1HM mice infected with CR6 exhibited aberrant responses to injury inflicted to the colon by dextran sodium sulfate (DSS), a treatment that induces a known suite of defects, including generation of ulcers in specific regions of the colon and loss of epithelial integrity in immunocompetent mice. While DSS treatment of uninfected ATG16L1HM and infected WT mice influences the colon comparable to that of littermate control mice, ATG16L1HM mice infected with CR6 seven days prior to DSS treatment are significantly more susceptible to DSS treatment. Treatment with blocking antibodies against tumor necrosis factor alpha (TNFα) and IFNG reduces the virus-plus-susceptibility gene phenotype as does, strikingly, treatment with broad-spectrum antibiotics [52]. The antibiotic responsiveness of this model reveals a bacterial component in the virus-plus-susceptibility gene interaction. With a characterized role for ATG16L1 in autophagy and pathogen control, this also implicates ATG16L1 and MNV in interaction with the murine microbiome [53,54,55].

The specific intersections between autophagy and viruses are still poorly understood on a broad scale. Further characterization of viral interaction with autophagy proteins as well as autophagy in general are necessary to create a clear picture of the possible outcomes. Several studies described here have posited a similar proviral role of autophagy for a wide range of RNA viruses, while for other viruses, evidence for both a proviral and antiviral role of autophagy exists [25,33]. It is likely that, as with other intracellular processes with which a virus must interact to successfully replicate, coevolution between the virus and the autophagy pathway has yielded an intricate network of interactions that cannot easily be described as strictly proviral or antiviral. Current evidence suggests that norovirus may benefit from the induction of autophagosome formation in some capacity, yet a noncanonical function of the LC3 conjugation system of autophagy with the IFN-inducible GTPases hinders MNV replication at the step of RC formation [4]. Future studies are required for complete understanding of this process, including most importantly, the manner by which ATG16L1 senses the RC and the mechanism of RC disruption by IRGs and GBPs.
